# Perceptions and acceptability of pictorial health warning labels vs text only - a cross-sectional study in Lao PDR

**DOI:** 10.1186/s12889-015-2415-9

**Published:** 2015-10-28

**Authors:** Vanphanom Sychareun, Visanou Hansana, Alongkone Phengsavanh, Kongmany Chaleunvong, Tanja Tomson

**Affiliations:** University of Health Sciences, Faculty of Postgraduate Studies, Vientiane, Lao PDR; Department of Learning, Informatics, Management and Ethics, (LIME), Karolinska Institutet, Stockholm, Sweden; Faculty of Postgraduate Studies and Research University of Sciences Vientiane, P.O. Box 744, Vientiane, Lao PDR

**Keywords:** Tobacco, Smoking, Health warnings, Awareness, Policy makers, Lao PDR

## Abstract

**Background:**

In Lao PDR, health warnings were first introduced with printed warning messages on the side of the cigarette package in 1993 and again in 2004. Lao PDR same year ratified the Framework Convention on Tobacco Control (WHO FCTC) but has not yet implemented pictorial health warnings. This paper aims to examine the perception and opinion of policymakers on “text-only” and “pictorial” health warnings and to understand lay people’s perceptions on current health warnings and their opinions on the recommended types of health warnings.

**Methods:**

A combination of quantitative and qualitative methods were used in this cross-sectional study conducted in 2008. A purposive sample of 15 policymakers, and a representative sample of 1360 smokers and non-smokers were recruited. A range of different areas were covered including consumer attitudes towards current and proposed cigarette package design, views on health warning messages on the flip/slide and inserts, and views on the relative importance of the size, content and pictures of health warning messages. Descriptive statistics and content analysis were used.

**Results:**

Policy makers and survey respondents said that the current health warning messages were inappropriate, ineffective, and too small in size. All respondents perceived pictorial health warnings as a potentially powerful element that could be added to the messages that can communicate quickly, and dramatically. The majority of policymakers and survey respondents strongly supported the implementation of pictorial health warnings.

The non-smokers agreed that the graphic pictorial health warnings were generally more likely than written health warnings to stimulate thinking about the health risks of smoking, by conveying potential health effects, increasing and reinforcing awareness of the negative health effect of smoking, aiding memorability of the health effects and arousing fear of smoking among smokers.

**Conclusions:**

The study suggested that current warnings are too small and that content is inadequate and designed to be hidden on the side pack. These findings are in line with FCTC’s requirements and provide strong support for introducing pictorial warning labels also in Lao PDR. Furthermore, the awareness of Members of Parliament about tobacco control measures holds promise at the highest political level.

**Electronic supplementary material:**

The online version of this article (doi:10.1186/s12889-015-2415-9) contains supplementary material, which is available to authorized users.

## Background

In Lao People’s Democratic Republic (Lao PDR), health warnings were first introduced in the country in 1993 with printed warning messages on the sides of the package of the cigarettes in the Lao language. The first brand of cigarettes was “Laimthong” followed by “Dok MaiDaeng”, whith the health warnings in English. The Marlboro and L&M cigarettes carried health warnings in both the Lao and English languages at the bottom of the front of the package. There are two big tobacco companies in Lao PDR, namely Lao-China Lucky Tobacco Company Limited and Lao Tobacco Company Limited, producing the two most popular brands of cigarettes: A Deng and Dok MaiDaeng (Red Flower) [1]. From 2003 to 2014, the Ministry of Health (MoH) also developed health warnings describing the hazards of smoking such as “Smoking is dangerous to health” in Lao and “do not sell tobacco to children under 18 years old” which were printed on the cigarette brand “555”. The aim of health warnings on tobacco products was to provide information about the health risks of smoking, the benefits of quitting, and to motivate people to quit [2].

**Fig. 1:**
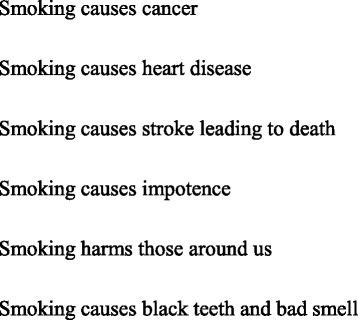
Current Health Message of Health warnings

In June 2004, Lao PDR ratified the World Health Organization (WHO) Framework Convention on Tobacco Control (FCTC), the first global tobacco control treaty. According to the FCTC, health warnings are required. Specifically, Article 11 states that the health warnings should be large, clear, visible, legible, and “should” cover 50 % or more, but no less than 30 % of the total display areas and may include pictures [3]. According to the labeling of Tobacco Product Containers Regulations, the health warnings on cigarette packages must be in Lao language and 6 warnings (Fig. 1) are to be carried in rotations approved by the Ministry of Health, (MOH) No 660/MOH dated 23 November, 2006 [4, 5].

According to the Tobacco Law endorsed in 2009, labels are texts determined by the government and printed on front and back sides of each packet, parcel, carton and case to show tobacco users that this product is dangerous and harmful to human health [6]. The tobacco companies in Lao PDR continue to ignore the health warning regulations. While the deadline for Lao to comply with the FCTC Article 11 was December 2009, Lao is pushing for a decree to help enforce the tobacco companies’ compliance with the regulations. These warnings were to be displayed in bold, white, letters in Saysetha Lao font with a size of 20, and on a black background covering the lower 30 % of the two largest surfaces of the packet. In the case of imported cigarettes, this should follow the current regulations of Lao PDR, should include key messages, and should cover 50 % or more of the displayed areas. More than 50 countries have adopted the FCTC recommendation for pictorial warnings that cover at least half of the package [7], but not Lao PDR [4].

Countries such as Canada, Brazil, Poland and Australia have already introduced graphic warnings through new legislations in this area, using more prominent messages and pictorial images. Previous research has suggested the role of on-pack messages as a valid health communication tool. A survey carried out in Australia [8] reported that health messages on cigarette packs resulted in an increased probability that the warning is noticed, and made smokers more likely to consider negative consequences.

Pictorial warnings are also necessary, particularly in countries with low literacy rates or where research shows smokers are ignoring standard warning labels. In Lao PDR the smoking prevalence among males is 43.1 %, and for females 8.4 %. The prevalence of smokeless tobacco was 7.9 % among female adults and 1 % for male adults [9]. Previous studies have shown that smokers in countries where a warning depicts a particular health hazard of smoking (e.g., impotence) are much more likely to know about such hazards [10].

Health warnings are important because they play a role in educating and informing smokers, especially young smokers, of the health risks of smoking (Elliot and Shanahan. Evaluation of the Health Warning Labels on Tobacco Products and Evaluation of the Commonwealth’s Information Line, prepared for Drugs of Dependence Branch, Commonwealth Department of Health and Family Services. unpublished). Previous studies highlight the potential of on-pack health information to inform smokers of the hazards of smoking, and to encourage quitting and to disrupt tobacco brand imagery [11–13]. Thus, the principle of large, picture-based warnings has been accepted on five continents. Amongst other advantages such warnings allow the chance to reach more vulnerable groups including women and children of smokers [14].

In order to minimize the disease caused by smoking, efforts need to be taken to make people aware of the consequences of smoking such as lung cancer, heart disease, stroke and to ensure that the most effective method is chosen to do this. Hence health warnings need to be designed and tested in order to recommend effective health warnings to the health policymakers. In addition, new regulation of health warnings describing the effect of tobacco use and pictorial warnings on all of tobacco products should be implemented in order to generate awareness of the hazards of smoking.

To our knowledge, this is the first study on cigarette package health warnings in Lao PDR. Our aim was to examine perceptions and of, and responses of pictorial and text health warnings among policymakers, members of parliament and the general public. The specific aims of this study were to: 1) describe perceptions of current text-only health warnings by smoking status, 2) examine the perceived effect of text-only health warnings by smoking status, and 3) to assess the perceived effectiveness of the pictorial health warnings as compared to text-only warnings.

## Methods

### Setting/study site

Lao PDR (Laos), a lower middle-income country, has 17 administrative provinces, one capital city and a population of 6,7 million. Target focus groups and testing were conducted in the Vientiane Capital City. The main reason for selecting this study site is that many government and non-government facilities and the National Assemblies are concentrated in Vientiane. In addition, there are many manufacturing factories, including those of the tobacco industry. Moreover, there were also some data available of the prevalence of smoking in Vientiane Capital city, which consisted of 4 urban districts (Chanthabury, Saysetha, Sisattanak, Sikhottabong) and 5 semi-urban and rural districts (Xaythany, Naxaythong, Hadxayphong, Santhong, Pakgnum). The Sikhottabong and Chanthabury districts were randomly selected as urban districts and Xaythany and Naxaythong districts were randomly included as semi-urban and rural districts.

This mixed-method study combined quantitative and qualitative research and covered a range of topics including consumer attitudes towards current and proposed cigarette package design, views on health warning messages on the flip/slide and inserts, and views on the relative importance of the size, content and pictures of health warning messages. Two different sub-studies were conducted: i) exploring the perceptions of policy makers; and ii) a survey of smokers and non-smokers.

### Data collection

#### In-depth interview of policymakers

A qualitative research methodology [15] was used to assess the perception and opinion of policymakers on health warnings on cigarette packs and the response of the pictorial design of tobacco health warnings. Qualitative in-depth interviews were used. The key informants were policymakers such as Members of Parliament who were chosen using purposive sampling. Initially, we recruited about 15 members of parliament from different ministries such as health, education, agriculture, culture, commerce and trade, and finance. However, the parliament members were not representatives of the ministries but were more representative of their provinces. In total, 15 parliament members from 14 out of 17 provinces were recruited for the study, with the exception of parliaments from Lunagnamtha, Champassack and Khammouane provinces. The key-informants were interviewed in Vientiane Capital during their Assembly meeting and they were asked about their opinion and attitudes regarding current health warnings, the most effective way of labelling tobacco to discourage smoking, and their opinion regarding the printed pictorial health warnings.

#### Survey of smokers and Non-smokers-Lay people

The aim of the survey of smokers and non- smokers was to capture their understanding of current health warnings and their view of the preferable type of health warnings, including format, colour and graphics, position and coverage, rotation and inserts, and other information (Additional file 1).

The target groups were adults aged 15–55 years of age. In total, 1360 participants were recruited (Table 1). They were selected in public places such as shopping centres, public parks, markets, restaurants, sporting venues, and entertainment venues. This method allowed us to approach target groups from different backgrounds. We changed location daily when recruiting participants. The respondents were approached privately, the purpose of the study was explained to them and they were then invited to participate in the study.

**Table 1:** Socio-demographic characteristics of respondents by smoking status

	Non-Smoker		Smoker		Total		*P* value
Variables	*N*	%	*N*	%	*N*	%	
Age (Mean = 32.42, SD = 13.68; Min-15, Max-55)							0.327
15-20	264	34.4	197	33.2	461	33.9	
21-35	205	26.7	143	24.1	348	25.6	
36-55	298	38.9	253	42.7	551	40.5	
Gender							<.001
Male	432	56.3	523	88.2	955	70.2	
Female	335	43.7	70	11.8	405	29.8	
Education level							<.001
No schooling	4	0.5	14	2.4	18	1.3	
Lower elementary	11	1.4	14	2.4	25	1.8	
Upper elementary	80	10.4	53	8.9	133	9.8	
Lower secondary	106	13.8	118	19.9	224	16.5	
Upper secondary	249	32.5	208	35.1	457	33.6	
Pre-university	114	14.9	73	12.3	187	13.8	
Diploma	56	7.3	41	6.9	97	7.1	
Bachelor	135	17.6	67	11.3	202	14.8	
Masters, PhD	12	1.6	5	0.8	17	1.3	
Occupation							<.001
Student	273	35.6	134	22.6	407	29.9	
Private Officer	71	9.3	64	10.8	135	9.9	
Government officer	120	15.6	72	12.1	192	14.1	
Farmer	19	2.5	22	3.7	41	3.0	
Housewife	31	4.0	10	1.7	41	3.0	
Owner enterprise	39	5.1	31	5.2	70	5.1	
Merchandise	114	14.9	61	10.3	175	12.9	
Daily paid worker	61	8.0	149	25.1	210	15.4	
Unemployed	23	3.0	32	5.4	55	4.1	
Driver	9	1.2	12	2.0	21	1.6	
Other	7	0.6	6	0.8	13	1.0	

The quantitative questionnaires included a brief section on socio-demographic background, followed by questions about perceptions of existing health warnings, as well as pictorial health warnings, the size of these warnings and key messages they contain. The guideline for interviewing policy makers composed of the socio-demographic characteristics, perception of existing health warnings and the future implementation of pictorial health warnings in Laos. To get the views of the participants on pictorial warnings, the research team showed them 10 mock packs adapted from different countries such as Thailand, Vietnam and other countries.

#### Data management, analysis, dissemination, and limitations

Data from all forms were entered into a standard relational database Epi.Info 6.0 and then transferred to SPSS 11.0. Data entry validity and integrity checks were performed by the data management team. Summary data results and quality assurance reports were forwarded to the field investigators. Data analysis consisted of descriptive statistics such as frequency, percentage, mean and SD, and some inferential statistics. Background information was run for univariate analysis. For comparing characteristics between smokers and non-smokers, categorical data were analyzed by using either Chi-Square or Fisher’s Exact Test. Multiple logistic regression were performed to test the observed differences between smokers and non-smoker in -awareness of health warnings on the side and back of packages and -perceived effectiveness of pictorial health warnings as compared to text-only warnings. This was done in order to control for confounding factors. Socio-demographic variables (age, sex, education, occupation) were adjusted for all analyses.

The survey questions and interviews were conducted in Lao language and then, the verbatim quote from the in-depth interviews were translated into English. The field notes were fully transcribed and then analyzed by two researchers. A content analysis technique was used to analyze the data [16, 17]. The analysis encompassed a back and forth process including an initial descriptive phase of identifying the meaning units and assigning codes to these. Then, the coding was compared and grouped into categories. The core of the qualitative data analysis is aimed towards systematization to identify themes, categories and codes and possible explanations for these themes.

After data collection the findings were disseminated to the stakeholders, especially to the National Committee Control for Tobacco. The findings from this study were used to strengthen health warnings on cigarette packages.

#### Ethical requirements

##### Informed consent of respondents

Ethical clearance was obtained from the National Ethical Review Board for Research, Ministry of Health. Verbal Informed consent was obtained from all respondents who answered the questionnaire or participated in the focus group discussions and interviews. In the Lao context the custom and ethics committee regulation is verbal informed consent for studies of this nature.

## Results

### Socio-demographic characteristics

#### In-depth interview of the members of parliament

A total of 15 members of parliament were interviewed. Half of the participants were female and participants represented nearly all the provinces in Lao PDR, except Luangnamtha, Champassack and Khammouane. Age ranged between 42 and 62 years and a few held more than one position in their province. Regarding their smoking status, the majority of males smoked compared to the female participants (34 % vs 1 %).

#### Survey of smokers and Non-smokers – Lay people

The socio-demographic characteristics of the participants are presented in Table 1. Overall, 1360 respondents were recruited. The age ranged from 15 to 55 years with a mean of 32.4 and SD = 13.7. About one third of respondents were female; while the vast majority of smokers were males (29.8 % vs 70.2 %).

One third of respondents (33.6 %) had completed upper secondary school, 16.5 % finished lower secondary schools and 14.9 % had received bachelor degrees (see Table 1). Non-smokers had a higher level of education (*p* < .001).

One third of the respondents were students (29,9 %), 15.4 % were daily paid workers and 14.1 % were government officers. The occupation of respondents was associated with smoking status, and daily paid workers were found to have smoked more than those in other occupations (*p* < .001).

### Smoking status

Table 2 illustrates smoking status by gender. Among the surveyed respondents, 44.6 % never smoked; 11.8 % were ex-smokers and 43.6 % were smokers with 25.4 % being daily smokers. There was a gender difference between smoking status with more daily smokers found among males compared to females (32.6 % versus 8.6 %, *p* < .001).

**Table 2:** Smoking status of respondents by gender

Variables	Male		Female		Total		*P* value
	*N*	%	*N*	%	*N*	%	
Smoking status							<.001
Never smoked	303	31.7	304	75.1	607	44.6	
Ex-smoker	129	13.5	31	7.7	160	11.8	
Occasional smoker	212	22.2	35	8.6	247	18.2	
Daily smoker	311	32.6	35	8.6	346	25.4	
Total	955	100	405	100	1360	100.0	

### Awareness/opinions of current health warnings

The study revealed that awareness of health messages on the front and back of cigarette package was quite low (Table 3). There was no statistical difference in awareness of the health messages between smokers and non smokers (19.2 % versus 18.1 %, *p* = .613). Awareness of health information on the side of cigarette packs was higher than awareness of warnings on the back and front of the packs. Smokers were more aware of the health warning on the side of the packs than non-smokers (84.5 % versus 51.8 %, *p* < .001). Awareness of warnings on the back of packages was the lowest of the three, and in all cases smokers were more conscious of warnings than non-smokers (14.7 % versus 9.9 %, *p* = .020).

**Table 3:** Perceptions towards current text-only health warnings

Variables	Non-smokers		Smokers		Total		*P* value
	*N*	%	*N*	%	*N*	%	
Awareness of health warning							
Front of pack							.613
Yes	139	18.1	114	19.2	253	18.6	
No	627	81.9	479	80.8	1106	81.4	
Side of pack							<.001
Yes	397	51.8	501	84.5	898	66.0	
No	370	48.2	92	15.5	462	34.0	
Back of pack							.0200
Yes	76	9.9	87	14.7	163	12.0	
No	690	57.8	506	85.3	1196	88.0	

Overall, recall of information on the front and back of the pack of cigarettes tended to be lower for all subgroups than awareness of health warning on the side of the pack of cigarettes. There were statistically significant differences between the recall ability of health warnings on the side (84.5 % vs 51.8 %, *p* < .001) and back of pack (14.7 % vs 9.9 %, *p* < .001) among smokers and non smokers. In the multiple logistic regression analysis, awareness of health warnings on the side of the pack and back of pack remained statistically significant (Additional file 2: Table S1).

Key informants from the National Assembly found that the lettering in the packs was too small and that there was a lack of contrast with the white background. As such, the current health warnings were less noticeable than they should be. They suggested that the message should be produced in a style that complements the other features of the pack.

*“The letters are too small, you can barely see them” (Male non-smoker, 52 years)*

*“The writing is too small to start with – no one reads it.” (Male smoker, 54 years)*

*“I saw the small letter in the side of the packs with one line. If you are not so interested, you won’t read it” (Male smoker, 62 years old)*

*“It is a small text, you can’t see it” (Female non-smoker, 46 years old)*

The health message was perceived to be too general and not indicating the composition or ingredients of the cigarettes such as the nicotine and tar content. In addition, some participants also commented that the message was too short and did not explain the health effects of smoking.

The key informants felt that the current messages say nothing and had been unchanged for many years. The messages were not attractive and were the least memorable element of the pack; the warnings were printed on the side of the pack, so people barely noticed them.

*“The current health message is not attractive to the smokers; however, some smokers even rarely read the health messages” (Female, 52 years old)*

*“The warning covered too small an area and is located by the side of the pack” (Male non-smoker, 52 years old)*

Pertaining to the content of the messages, the current perception is that the present content was inadequate as it only has one sentence. Furthermore, the messages did not deal with specific diseases.

*“The information is scarce, and the content of the message is not in-depth and not adequate” (Male smoker, 56 years old)*

Concerning the effectiveness of the current health warnings, some considered warnings such as “Smoking is dangerous to your health” to be too general and thus would not encourage smokers to give up the habit:

*“The current health warnings are too general and the letters are too small, not attractive and not prominent, so you can’t quit smoking”. (Male smoker, 50 years old)*

However, many of the respondents agreed that the content of the health warning was credible.

*“I think, the health warnings are credible because it is true and there are smokers getting some diseases” (Male smoker, 54 years old)*

A few Members of Parliament mentioned that the messages were generally clear, short and the public likes simple and direct messages.

*“It is simple and does not go around” (Male, 52 years old).*

### Perceived effect of health warnings on knowledge

Table 4 shows the effect of health messages on knowledge of smoking and its impact on health. Approximately 13.7 % of respondents, irrespective of their smoking status, claimed that the health warnings have not raised their awareness about the health risks at all while 46.7 % indicated that they thought about health risks “a lot”. Compared to non-smokers, smokers were more likely to be concerned about health risks (*p* < .001).

**Table 4:** Perceived effect of current text-only health warnings on thoughts and knowledge about smoking harms

	Non-smoker		Smoker		Total	
Variables	*N*	%	*N*	%	*N*	%
Health warnings make you think about the health risks (*p* < .001)						
Not at all	99	13.1	86	14.6	185	13.7
A little	121	16.0	168	28.4	289	21.4
Somewhat	142	18.8	103	17.4	245	18.2
A lot	395	52.2	234	39.6	629	46.7
Inclusion of health warnings and health information on cigarette packs has improved your knowledge of the health effects of tobacco (*p* < .001)						
Not at all	70	9.2	82	13.9	152	11.3
A little	110	14.5	145	24.5	255	18.9
Somewhat	157	20.7	134	22.7	291	21.6
A lot	422	55.6	230	38.9	652	48.3

About 48.3 % of respondents suggested that their knowledge about the health effects of tobacco had improved “a lot” as a result of the inclusion of health warnings and health information on cigarette packs. Compared to non-smokers, smokers were more inclined to suggest that their knowledge had improved. After controlling for potential confounders, this remained statistically significant (Additional file 2: Table S2).

### Perceived effectiveness of pictorial health warnings

In comparison with “text only”, alternatives of the graphic pictorial health warnings were generally more likely to stimulate thinking about the health risks of smoking (82.9 %), with non-smokers giving more thought about the health risks compared to smokers (*p* < .001) (Table 5).

**Table 5:** Perceived effectiveness of pictorial health warnings as compared to text-only warnings

	Non- Smoke		Smoker		Total		*P*-value
Effectiveness of pictorial health warning	767		593				
	*N*	%	*N*	%	*N*	%	
a. In making you think of the health risk of smoking	669	87.3	458	77.2	1127	82.9	<.001
b. In conveying potential health effect of smoking effectively?	659	86.1	448	75.5	1107	81.5	<.001
c. In increasing and reinforcing awareness of the negative health effect of smoking?	629	83.0	450	76.1	1079	80.0	.008
d. In aiding memorability of the health effects?	631	82.5	442	74.5	1073	79.0	.001
e. In arousing fear of smoking	616	80.5	434	73.2	1050	77.3	.004
f. In encouraging smokers to quit?	419	54.8	334	58.2	763	56.3	.370
g. In encouraging smokers in general to think about their smoking habit?	546	71.5	394	66.8	940	69.4	.163

The pictorial health warnings more effectively conveyed the potential health effects (81.5 %); increasing and reinforcing awareness of the negative health effects of smoking (80 %); aiding memorability of the health effects (79 %); and arousing fear of smoking (77.3 %) with statistically significant differences between non-smokers and smokers (*P* < .001; *P* = .008 and *P* = .001 respectively). However, compared to “text only”, the pictorial health warnings were not effective in encouraging smokers to quit. The study revealed that only 56.3 % claimed that pictorial health warnings were more likely to encourage smokers to give up smoking; in addition, 69.4 % indicated that pictorial health warnings were more likely to encourage smokers to think about their smoking habits. However, there was no statistically significant difference between the smokers and non-smokers on the statements “encouraging to quit smoking” and “encouraging to think about their smoking habit” (*p* = .370 and *p* = .163 respectively) (Table 5). After controlling for potential confounders, this difference was statistically significant (Additional file 2: Table S3).

The qualitative findings also verified the quantitative research method. The pictorial health warnings were perceived to be more effective because smokers could see the pictures directly with messages, or even without messages. The graphic packs in particular tended to reinforce the decision of young nonsmokers to not consider or take up smoking.

*“I think it’s excellent because on a normal cigarette pack all they have is writing text such as smoking kills you or smoking is danger for your health and people around you. They don’t have any pictures, they don’t tell you what it actually does to your body. I think if we get the picture of lung cancer or other pictures with severe health consequences from smoking such as throat cancer and so on. And the pictures are more attractive and more persuasive.”* (*Female non smoker, 51 years*)

### Perception of the implementation of pictorial health warnings

About 65 % of respondents suggested that health warnings on tobacco packs were very important and 30.5 % cited health warnings were quite important. Non- smokers were more likely to rate the health warnings on tobacco and cigarette packs as “very important”, when compared to smokers (69.1 % versus 59.8 %, *p* = .005). With regards to the perceived effectiveness of pictorial health warnings, 44 % pointed out that pictorial health warnings would be effective; on the other hand, 19.4 % noted that pictorial health warnings would be neutral (neither effective nor ineffective). There was no statistically significant difference between effective pictorial health warnings and smoking status (*p* = .742). Approximately 65.2 % strongly supported implementation of graphic health warnings on cigarette packs with non-smokers more so than smokers (71.3 % versus 57.3 %, *p* < .001).

Similarly, for the qualitative findings, all policy makers supported the implementation of pictorial health warnings because people’s fear will be raised. Pictorial health warnings may not only make people less likely to smoke but also increase public awareness of the health hazards of using tobacco products.

*“I fully agree to implement the pictorial health warnings in our country because people will be more aware of the dangers of smoking; however, we need to explore more in-depth which of the health warnings and the pictorial that will be suitable for Laos.”* (Male, 52 years old)

However, some policy makers had some concerns about the implementation of pictorial health warnings as the majority of rural people use rolling tobacco, which obviously does not include pictorial health warnings.

Regarding the perceived effect of pictorial health warnings on knowledge, 89.3 % stated that knowledge of the effect of smoking would be improved due to the implementation of the tobacco policy; while non-smokers would be more likely to claim that their knowledge on the health effect would be improved, as compared to smokers (*p* < .001).

In relation to the size of pictorial health warnings, 42.2 % stated that the size of pictorial health warnings should be 50 % of the display area to be more effective. It is interesting to note that smokers were less likely to agree to have greater size displays on the tobacco packs than non-smokers (*p* < .001). Similarly, most policy makers agreed that the pictorial health warnings should be 20 to 50 % of the display areas of the pack and on the front of the pack, so people can notice them directly.

*“The size of health warning should be 50 % of the front pack because people who smoke will see immediately and the other side, it will be the key information of the harmful effect of smoking” (Male, aged 51 years)*

## Discussion

### Awareness of current health warning with “text only”

Our study is a response to a request from a systematic review regarding “health warning messages on tobacco products”, namely to include studies from low-income countries [12]. This type of research context is important and it is unclear whether the impact of messages varies a lot between high- and low- to middle-income countries [12]. This study revealed interesting findings on the awareness of health warning messages on the side of packs of cigarettes, with smokers being more likely to be aware of the health warnings than non-smokers.

Awareness of health warnings on the front and back of packs and readership of the health warnings was low and recall of health warnings was also low. The position of health warning on the side of packs made them less likely to be noticed than if they were on the front, a finding that was similar to the survey by European tobacco control organizations [18].

The perceived effect of “text only” health warnings was low, as indicated by a lower association with awareness of health risks, perceived likelihood of improving knowledge on health effects, and quitting.

Overall, the health warnings with “text only” was associated with a perception of not having an impact and with the perception of not being effective in conveying the potential negative health consequences of smoking. The results from this study were consistent with findings from previous research comparing reactions to text-only warning labels in other countries [19–21]. These authors also found that health knowledge is lower among smokers, even in highly educated countries; however, they would expect health knowledge to be substantially lower among the majority of the world’s smokers, particularly those living in lower and middle income countries. Thus, the effectiveness of health warning labels could address knowledge deficits by providing comprehensive health warnings to smokers without regular access to health information on the risks of smoking.

In regards to the effectiveness of current health warnings, only slightly higher than half of the respondents perceived that the health warnings with “text only” had some effectiveness. As the literature has suggested, warning labels with text-only did not have high effectiveness [22]. The study carried out by the European tobacco control organizations also showed that text-only health warnings are largely ignored by smokers because they are difficult to see and tend to blend in with the packing design [17]. Thus, the current health warning system has clearly failed to adequately inform people of the risks of smoking and needs to be changed.

The qualitative data from in-depth interviews of Members of Parliament also revealed that the current health warnings were perceived to be inadequate, less noticeable, less believable and provide less information than pictorial health warning. Thus, there is clearly the need to improve the format to make content more visually prominent, simulating, specific and persuasive. Most of the informants believed that the health warnings are outdated, do not include any new information and that the warnings have lost their novelty and noticeability. Similarly, the FCTC also claimed that to be effective, health warnings should be prominent enough to capture smoker’s’ attention and must break down the “wear-out” that results from habituation to the message. Thus the warning labels should be 50 % or more of the principal display areas, but not less than 30 % [23].

This study revealed that the policy makers suggested to have the size of health warnings to be 50 % to 100 % of the principle area of the cigarette package, which is consistent with the Framework of FCTC [14]. Given the exceptionally hazardous nature of tobacco and the failure of tobacco companies to adequately disclose risks, warnings should occupy at least as much area on tobacco product packaging as any artwork designed to make the package attractive. Recently, many countries have passed laws requiring that health messages comprise significant portions of the front and back of the package.

Moreover, the prevailing health warnings in Laos only covers 30 % of the packet and may not be impressive or useful enough to remind people of the hazards of smoking, and that “cigarettes are harmful not only to smokers but also to second-hand smokers,”. The Tobacco Control in Lao PDR aimed to improve health warnings in order to increase awareness of the harmful effects of smoking, and to reduce the rate of smoking among children. The health warnings on tobacco products have the following functions: to provide information about the health risks of smoking, to provide information on the benefits of quitting, to motivate people to quit, to deter people from starting to smoke or from becoming habitual users and to help those who have decided to quit to do so. However, these health warnings have not been implemented in Lao PDR yet.

### Effectiveness of pictorial health warnings

The study revealed that the vast majority of respondents believed in the effectiveness of pictorial health warnings. In comparison with the “text only” warnings the pictorial health warnings were generally thought to be more likely to convey potential health effects of smoking more effectively, to increase and reinforce awareness of the negative health effects of smoking, to aid memorability of the health effects, to encourage smokers to quit and to think about their smoking habits. All evidence from previous studies suggested that graphic warnings were (i) a prominent source of health information second only to television in many jurisdictions; (ii) more likely to be noticed and discussed than text warnings, (iii) associated with greater health knowledge, (iv) associated with increased cessation behavior, and (v) that these warnings enjoy high effectiveness and support from smokers themselves [21, 22]. These authors also found that the graphic will contribute to an increase in the unacceptability of smoking for both health and social reasons. In addition, the graphics will increase anxiety and anger and will elicit more emotional reaction [22].

This present study was consistent with previous studies indicating that prominent warning labels and more comprehensive health messages with graphic elements are more likely to both encourage smokers to forego smoking than text-only warning labels and to be noticed and cited as effective by smokers [21, 22, 24]. Previous surveys of smokers have been carried out in the USA, Canada, and the United Kingdom and Australia with widely different health warnings ranging from large graphic depictions of diseases in Canada to small text-only warnings in the USA. Smokers in our study were less likely to perceive the effect of pictorial health warnings in conveying the potential heath risks. This increases and reinforces awareness of the negative health effect of smoking. Smokers in Canada were the most likely to report thinking about the health risks of smoking, to stop having cigarettes and to think about quitting; however, smokers in the US reported the lowest levels of effectiveness for almost all measured items [21]. Smokers in countries where a warning depicts a particular health hazard of smoking are much more likely to know about the health hazards and smokers who reported noticing warnings were 1.5 to 3 times more likely to believe each health hazard [19].

Thus, the smokers in our study did not pay any attention to the current health warnings. The pictorial warnings are more likely to have impact, to attract attention, be confrontational to smokers and to be difficult to ignore. The study showed that pictorial health warnings have more impact on knowledge of the risk of smoking, and on quitting and help to convey potential health effects of smoking more effectively through pictures than words. The rationale for the potentially greater effectiveness of graphic health warnings when compared to text only, is that “a picture is worth a thousand words” and evokes an emotional response [9] raising fear and social stigma among smokers to increase their awareness and attract their attention.

### Perspective on the implementation of pictorial health warnings

Most policymakers strongly supported implementation of graphic health warnings on cigarette packs. The policymakers have a strong moral obligation to inform consumers about the risks of smoking. The primary intent of pictorial warnings is not to scare, but to inform smokers about the full range, likelihood, and severity of smoking-related diseases.

In contrast, some policymakers raised concerns about the implementation of pictorial health warnings within the Lao cultural context because the majority of rural people used rolling tobacco, thus they might not have access to health warning images. Parallel to this, there should be a focus on more effective interventions and policies such as dissemination of health information through various channels such as radio, posters, leaflets, health education in the community; and the integrating of health information into the school curriculum.

## Conclusion

The study findings suggest that in Lao PDR the current tobacco package warnings are too small, their content is inadequate to convey health risks, and their design is such that they are hidden in the side of the packages. These findings support the FCTC’s requirements for introducing pictorial warning labels in Lao PDR. Furthermore, the awareness of Members of Parliament about tobacco control measures holds promise at the highest political level.

## Additional files

Additional file 1:
**Survey Questionnaire for Perception of Cigarette Health Warnings in the Lao PDR.** (DOCX 75 kb) Additional file 2: Table S1: Multiple logistic regression in awareness of health warnings on side and back of packs between smokers and non-smokers. Table S2: Multiple logistic regression in Perceived effect of current text-only health warnings on thoughts and knowledge about smoking harms between smokers and non-smokers. Table 3: Multiple logistic regression in Perceived effectiveness of pictorial health warnings as compared to text-only warnings between smokers and non-smokers. (DOCX 22 kb)
