# Supplementary material for: Perceptions and acceptability of pictorial health warning labels vs text only - a cross-sectional study in Lao PDR
Source: BMC Public Health. 2015 Oct 28;15:1094. doi: 10.1186/s12889-015-2415-9 (PMC4625568; doi:10.1186/s12889-015-2415-9)
Supplement: Additional file 1: — Survey Questionnaire for Perception of Cigarette Health Warnings in the Lao PDR. (DOCX 75 kb) [file 12889_2015_2415_MOESM1_ESM.docx]

Survey Questionnaire for Perception of Cigarette Health Warnings in the Lao PDR Date:___________________________________ Name of interviewers______________________ Id number_______________________________ District_________________________________Province_____________________ Urban/Rural_____________________________

**I. Socio-demographic Characteristic:**

1.What is your age? ________________(Enter number)

2. What is your gender?

1 Male

2 Female

3. What is the highest level of formal education that you have completed?

1. No schooling

2. Lower elementary

3. Upper elementary

4. Lower secondary

5. Upper secondary

6. Pre-university

7. Diploma, certificate

8. Bachelor degree

9. Masters, PhD degree

10. Other -- Specify: __________________________

4. What is your main occupation?

1. Student

2. Private officers

3. Government Officers

4. Farmers

5. Housewife

6. Owner enterprise

7. Merchandise

8. Daily paid worker

9. Unemployment

10. Other --- Specify: __________________________

**II. Smoking status**

5. Which of the following best describes your smoking?

1.I have never smoked

2. I have quit smoking

3. I currently smoke at least a few times a week

4. I currently smoke everyday

**III. Perceptions towards health warning**

6. Are you aware of any health message on the front, side or the back of tobacco

pack?

6.1 Front of pack

1. Yes

2. No

3. DK

6.2. Side of pack

1. Yes

2. No

3. DK

6.3 Back of pack

1. Yes

2. No

3. DK

7. I’m going to read out to you some health messages and information. Could you

please tell me if the messages or information appears on the pack or does not

appear at all or if you are uncertain? (ROTATE & READ)

|  | 1.Yes | 2.No | 3.Uncertain |
| --- | --- | --- | --- |
| 1. Smoking causes heart disease |  |  |  |
| 2. Smoking causes lung cancer |  |  |  |
| 3. Smoking is addictive |  |  |  |
| 4. Smoking causes throat cancer |  |  |  |
| 5. Smoking reduces your health |  |  |  |
| 6. Smoking kills |  |  |  |
| 7. Smoking can harm others |  |  |  |
| 8. Smoking when pregnant harms your baby |  |  |  |
| 9. Smoking causes cancer of mouth |  |  |  |

8. In the last month, how often, if at all, have you read or looked closely at the health

warnings on cigarette packages?

1. Never

2. Once

3. A few times

4. Many times

9. In the last month, have the health warnings stopped you from having a cigarette

when you were about to smoke one? Would you say . . . (For smokers)

1. Never

2. Once

3. A few times

4. Many times

10. To what extent, if at all, do the health warnings make you think about the health

risks (health dangers) of smoking?

1. Not at all

2. A little

3. Somewhat

4. A lot

11. Would you say the inclusion of health warnings and health information on

cigarette packs has improved your knowledge of the health effects of tobacco

consumption?

1. Not at all

2. A little

3. Somewhat

4. A lot

12. To what extent, if at all, do the health warnings on cigarette packs make you more

likely to quit smoking? (For smokers)

1. Not at all

2. A little

3. Somewhat

4. A lot

13. In terms of the way you feel about your own smoking behavior would you say the

health warnings on packs of cigarettes and tobacco have helped you smoke less?

(For smokers)

1. Not at all

2. A little

3. Somewhat

4. A lot

14. Would you say that the health warning information on cigarette packages is:

1. Not at all

2. A little

3. Somewhat

4. A lot

15. Do you think that cigarette packages should have more health information than

they do now, less, or about the same amount as they do now?

1. Less health information

2. About the same

3. More health information

16. Note: Show a cigarette pack with “text only” warning and a sample of a cigarette

pack with graphic warning.

| (Compared to ”text only” warning, is the graphic warning more likely, less likely or make no difference .....) | Tick ☑ in the appropriate box | | |
| --- | --- | --- | --- |
|  | **^1^More likely** | **^2^Less likely** | **^8^Make no difference** |
| a. In making you think of the health risk of smoking | 🞎 | 🞎 | 🞎 |
| b. In conveying potential health effect of smoking effectively? | 🞎 | 🞎 | 🞎 |
| c. In increasing and reinforcing awareness of the positive health effect of smoking? | 🞎 | 🞎 | 🞎 |
| d. In aiding memorability of the health effects? | 🞎 | 🞎 | 🞎 |
| e. In arousing fear of smoking | 🞎 | 🞎 | 🞎 |
| f. In encourageing smokers to quit? | 🞎 | 🞎 | 🞎 |
| g. In encouraging smokers in general to think about their smoking habit? | 🞎 | 🞎 | 🞎 |

17. How important is it that the Government has health warnings on packs of tobacco

and cigarettes. Would you say...

1. Very important

2. Quite important

3. Neither important

4. Quite unimportant

5. Very unimportant

18. Do you think printing pictorial health warnings about harmful effects of smoking

on cigarette packs is an effective way to reduce smoking among young people?

1. Very effective

2. Effective

3. Neither effective nor ineffective

4. Ineffective

5. Very ineffective

19. Do you support the implementation of graphic health warnings on cigarette packs

in the Lao PDR?

1. Strongly support

2. Somewhat support

3. Neither support nor oppose

4. Strongly oppose

5. Somewhat oppose

20. Would you say the inclusion of pictorial health warnings and health

information on cigarette packs has improved your knowledge of the

health effects of tobacco consumption...

1. A lot

2. A little

3. Made no difference

4. Don’t know

21.Which size of pictorial health warning is more effective?

1. 25%

2. 30%

3. 50%

4. 70%

Thnak you for your cooperation.
