# Supplementary material for: Perceptions and acceptability of pictorial health warning labels vs text only - a cross-sectional study in Lao PDR
Source: BMC Public Health. 2015 Oct 28;15:1094. doi: 10.1186/s12889-015-2415-9 (PMC4625568; doi:10.1186/s12889-015-2415-9)
Supplement: Additional file 2: Table S1: — Multiple logistic regression in awareness of health warnings on side and back of packs between smokers and non-smokers. Table S2: Multiple logistic regression in Perceived effect of current text-only health warnings on thoughts and knowledge about smoking harms between smokers and non-smokers. Table 3: Multiple logistic regression in Perceived effectiveness of pictorial health warnings as compared to text-only warnings between smokers and non-smokers. (DOCX 22 kb) [file 12889_2015_2415_MOESM2_ESM.docx]

Additional file 2

Table S1: Multiple logistic regression in awareness of health warnings on side and back of packs between smokers and non-smokers.

| **Variables** | **Smoking** | | **Adjusted OR** | **95% CI** | **P value** |
| --- | --- | --- | --- | --- | --- |
|  | N | % |  |  |  |
| **Age** |  |  |  |  |  |
| 15-20 | 197 | 42.7 | 1 |  |  |
| 21-35 | 143 | 41.1 | 0.9 | 0.6 – 1.2 | 0.342 |
| 36-55 | 253 | 253 | 1.1 | 0.8 – 1.5 | 0.494 |
| **Gender** |  |  |  |  |  |
| Male | 523 | 54.8 | 1 |  |  |
| Female | 70 | 17.3 | 0.2 | 0.1 – 0.2 | < 0.001 |
| **Education** |  |  |  |  |  |
| Noschool-Elementary | 81 | 46.0 | 1 |  |  |
| Highschool to Pre-University | 399 | 46.0 | 1.1 | 0.8 – 1.6 | 0.629 |
| University & Post-University | 113 | 35.8 | 0.9 | 0.6 – 1.3 | 0.520 |
| **Occupation** |  |  |  |  |  |
| Student/Officer | 270 | 36.8 | 1 |  |  |
| Farmer/Housewives | 32 | 39.0 | 1.2 | 0.7 – 2.0 | 0.519 |
| Entreprise/Merchandises | 92 | 37.6 | 0.9 | 0.7 – 1.3 | 0.678 |
| Dailyworker/Driver | 161 | 69.7 | 4.4 | 3.0 – 6.3 | < 0.001 |
| Others (Unemployed & others) | 38 | 55.9 | 2.3 | 1.3 – 4.2 | 0.004 |
| **Awareness of HW Front of pack** |  |  |  |  |  |
| No | 479 | 43.3 | 1 |  |  |
| Yes | 114 | 45.1 | 1.0 | 0.7 – 1.4 | 0.996 |
| **Awareness of HW Side of pack** |  |  |  |  |  |
| No | 92 | 19.9 | 1 |  |  |
| Yes | 501 | 55.8 | 5.2 | 3.9 – 6.9 | <0.001 |
| **Awareness of HW Back of pack** |  |  |  |  |  |
| No | 506 | 42.3 | 1 |  |  |
| Yes | 87 | 53.4 | 1.5 | 1.0 – 2.2 | 0.038 |

Table S2: Multiple logistic regression in **Perceived effect of current text-only health warnings on thoughts and knowledge about smoking harms** between smokers and non-smokers.

| **Variables** | **Smoking** | | **Adjusted OR** | **95% CI** | **P value** |
| --- | --- | --- | --- | --- | --- |
|  | N | % |  |  |  |
| **Age** |  |  |  |  |  |
| 15-20 | 197 | 42.7 | 1 |  |  |
| 21-35 | 143 | 41.1 | 0.9 | 0.6 – 1.2 | 0.389 |
| 36-55 | 253 | 253 | 1.1 | 0.8 – 1.4 | 0.570 |
| **Gender** |  |  |  |  |  |
| Male | 523 | 54.8 | 1 |  |  |
| Female | 70 | 17.3 | 0.2 | 0.1 – 0.2 | < 0.001 |
| **Education** |  |  |  |  |  |
| Noschool-Elementary | 81 | 46.0 | 1 |  |  |
| Highschool to Pre-University | 399 | 46.0 | 1.1 | 0.7 – 1.5 | 0.757 |
| University & Post-University | 113 | 35.8 | 0.7 | 0.5 – 1.0 | 0.117 |
| **Occupation** |  |  |  |  |  |
| Student/Officer | 270 | 36.8 | 1 |  |  |
| Farmer/Housewives | 32 | 39.0 | 1.2 | 0.7 – 2.0 | 0.538 |
| Entreprise/Merchandises | 92 | 37.6 | 1.0 | 0.7 – 1.3 | 0.855 |
| Dailyworker/Driver | 161 | 69.7 | 4.2 | 2.9 – 5.9 | < 0.001 |
| Others (Unemployed & others) | 38 | 55.9 | 2.3 | 1.3 – 4.0 | 0.003 |
| **Health warnings make you think about the health risks** | | | | | |
| Not at all-A little | 255 | 53.8 | 1 |  |  |
| Somewhat-A lot | 338 | 38.7 | 0.5 | 0.4 – 0.7 | < 0.001 |
| **Inclusion of health warnings and health information on cigarette packs has improved your knowledge of the health effects of tobacco** | | | | | |
| Not at all-A little | 227 | 55.8 | 1 |  |  |
| Somewhat-A lot | 366 | 38.8 | 0.5 | 0.4 – 0.6 | < 0.001 |

Table S3: Multiple logistic regression in **Perceived effectiveness of pictorial health warnings as compared to text-only warnings** between smokers and non-smokers.

| **Variables** | **Smoking** | | **Adjusted OR** | **95% CI** | **P value** |
| --- | --- | --- | --- | --- | --- |
|  | N | % |  |  |  |
| **Age** |  |  |  |  |  |
| 15-20 | 197 | 42.7 | 1 |  |  |
| 21-35 | 143 | 41.1 | 0.9 | 0.6 – 1.2 | 0.435 |
| 36-55 | 253 | 253 | 1.1 | 0.9 – 1.5 | 0.367 |
| **Gender** |  |  |  |  |  |
| Male | 523 | 54.8 | 1 |  |  |
| Female | 70 | 17.3 | 0.2 | 0.1 – 0.2 | < 0.001 |
| **Education** |  |  |  |  |  |
| Noschool-Elementary | 81 | 46.0 | 1 |  |  |
| Highschool to Pre-University | 399 | 46.0 | 1.0 | 0.7 – 1.4 | 0.993 |
| University & Post-University | 113 | 35.8 | 0.7 | 0.5 – 1.1 | 0.087 |
| **Occupation** |  |  |  |  |  |
| Student/Officer | 270 | 36.8 | 1 |  |  |
| Farmer/Housewives | 32 | 39.0 | 1.0 | 0.6 – 1.7 | 0.914 |
| Entreprise/Merchandises | 92 | 37.6 | 0.9 | 0.7 – 1.3 | 0.739 |
| Dailyworker/Driver | 161 | 69.7 | 4.1 | 2.9 – 5.8 | < 0.001 |
| Others (Unemployed & others) | 38 | 55.9 | 1.9 | 1.1 – 3.4 | 0.020 |
| a. In making you think of the health risk of smoking | 458 | 40.6 | 0.5 | 0.4 – 0.7 | < 0.001 |
| b. In conveying potential health effect of smoking effectively? | 448 | 40.5 | 0.6 | 0.4 – 0.8 | < 0.001 |
| c. In increasing and reinforcing  awareness of the negative health effect of smoking? | 450 | 41.7 | 0.7 | 0.5 – 0.9 | 0.018 |
| d. In aiding memorability of the health effects? | 442 | 41.2 | 0.6 | 0.5 – 0.8 | 0.001 |
| e. In arousing fear of smoking | 434 | 41.3 | 0.6 | 0.5 – 0.9 | 0.002 |
| f. In encouraging smokers to quit? | 334 | 44.4 | 1.0 | 0.8 – 1.3 | 1.000 |
| g. In encouraging smokers in general to think about their smoking habit? | 394 | 41.9 | 0.8 | 0.6 – 1.1 | 0.069 |
